# Efficacy of an integrative approach for bipolar disorder: preliminary results from a randomized controlled trial

**DOI:** 10.1017/S0033291721001057

**Published:** 2022-12

**Authors:** Èlia Valls, C. Mar Bonnín, Imma Torres, Mercè Brat, Mireia Prime-Tous, Ivette Morilla, Xavier Segú, Brisa Solé, Carla Torrent, Eduard Vieta, Anabel Martínez-Arán, María Reinares, José Sánchez-Moreno

**Affiliations:** 1 Barcelona Bipolar Disorders and Depressive Unit, Institute of Neurosciences, University of Barcelona, IDIBAPS, CIBERSAM, Hospital Clínic of Barcelona, Villarroel, 170, 08036 Barcelona, Catalonia, Spain; 2 Department of Psychiatry and Psychology, Institute of Neurosciences, Hospital Clínic of Barcelona, Villarroel, 170, 08036 Barcelona, Catalonia, Spain

**Keywords:** Bipolar disorder, functional remediation, integrative approach, mindfulness, psychoeducation, psychological treatment

## Abstract

**Background:**

Bipolar disorder (BD) represents one of the most therapeutically complex psychiatric disorders. The development of a feasible comprehensive psychological approach to complement pharmacotherapy to improve its clinical management is required. The main objective of the present randomized controlled trial (RCT) was to test the efficacy of a novel adjunctive treatment entitled integrative approach in patients with BD, including: psychoeducation, mindfulness training, and functional remediation.

**Methods:**

This is a parallel two-armed, rater-blind RCT of an integrative approach plus treatment as usual (TAU), *v.* TAU alone. Participants were recruited at the Hospital Clinic of Barcelona and randomized to one of the two conditions. They were assessed at baseline and after finishing the intervention. The main outcome variable included changes in psychosocial functioning assessed through the Functioning Assessment Short Test (FAST).

**Results:**

After finishing the treatment, the repeated-measures analyses revealed a significant group × time interaction in favor of the patients who received the integrative approach (*n* = 28) compared to the TAU group (*n* = 37) (Pillai's trace = 0.10; *F*_(1,57)_ = 6.9; *p* = 0.01), improving the functional outcome. Significant effects were also found in two out of the six domains of the FAST, including the cognitive domain (Pillai's trace = 0.25; *F*_(1,57)_ = 19.1; *p* < 0.001) and leisure time (Pillai's trace = 0.11; *F*_(1,57)_ = 7.15; *p* = 0.01). Regarding the secondary outcomes, a significant group × time interaction in Hamilton Depression Rating Scale changes was detected (Pillai's trace = 0.08; *F*_(1,62)_ = 5.6; *p* = 0.02).

**Conclusion:**

This preliminary study suggests that the integrative approach represents a promising cost-effective therapy to improve psychosocial functioning and residual depressive symptoms in patients suffering from BD.

## Introduction

Bipolar disorder (BD) is a lifelong mental condition characterized by recurrent mood episodes, persistent symptoms, and potential cognitive and psychosocial impairment, which may affect patients' well-being and quality of life (Carvalho, Firth, & Vieta, 2020). It represents one of the most therapeutically complex psychiatric disorders (Vieta et al., 2018). Even though pharmacological treatment is essential in the clinical management of BD (Yatham et al., 2018), it is often not enough to reach complete clinical and functional recovery and improve quality of life (Vieta et al., 2012, 2013). In this regard, complementary psychological interventions are necessary as they play a crucial role in the self-management of the disease; covering aspects that medication alone cannot achieve (Reinares, Sánchez-Moreno, & Fountoulakis, 2014). A significant number of psychological interventions have been developed and tested in the field of BD over the last two decades. Most of the initial treatments were developed with the objective to improve pharmacological adherence and prevent relapses. Among these therapies, psychoeducation has proven its efficacy at increasing the time and the risk of relapses and hospitalizations (Colom et al., 2003, 2009). Family interventions have also proved their efficacy at reducing the risk of relapses and increasing time to relapse (Miklowitz, George, Richards, Simoneau, & Suddath, 2003; Miklowitz et al., 2000; Reinares et al., 2008, 2010), as well as having a positive impact on family caregivers (Reinares et al., 2016). Positive findings in terms of relapse prevention have been reported with cognitive-behavioral therapy (Lam et al., 2003), particularly in patients with less severity (Scott et al., 2006). Other treatments, such as the Interpersonal and Social Rhythm Therapy suggested that engaging into regular habits and regularity of social rhythms could speed up the improvement of occupational functioning (Frank et al., 2008) and mood symptoms (Inder et al., 2017; Swartz et al., 2017).

However, in the last few years the design of psychological interventions has broadened its objectives beyond preventing relapses and reducing symptoms, as they include other targets such as improving psychosocial functioning, quality of life, and even enhancing cognitive functioning (Bonnín et al., 2019). The interest in psychosocial functioning might be driven mainly because early in the beginning of the 2000s a growing body of evidence started to point out that patients with BD suffered from cognitive deficits that compromised seriously their functional outcome (Bonnín et al., 2010; Martínez-Arán et al., 2004, 2007; Tabarés-Seisdedos et al., 2008). Indeed, it was the beginning of the cognitive/functional remediation programs (Bonnin, Torrent, Vieta, & Martínez-Arán, 2014; Martínez-Arán, 2011) that aimed to improve psychosocial functioning and cognitive skills; nevertheless, results from these therapies are still inconclusive, and probably replication of findings is needed. Recently, a systematic review indicated that efficacy of functional remediation was moderate, with effect sizes around 0.45 pointing at significant benefits for psychosocial functioning (Tsapekos et al., 2020).

Given that therapies developed so far tackle separately different needs of the disease (adherence, improving symptoms, improving functioning, etc.), the development of a comprehensive psychological approach to complement pharmacotherapy and that include several targets is required. In this regard, an intervention that combines the main components of different treatments in a smaller number of sessions to make it briefer, simpler and widespread, encompassing broader therapeutic outcomes and improving the prognosis of BD disease is urgently needed.

That is why the Bipolar and Depressive Disorders Unit from the Hospital Clinic has recently developed an adjunctive integrative approach (Reinares, Martínez-Arán, & Vieta, 2019) consisting of 12 weekly closed-group sessions for patients with BD. This comprehensive approach combines therapeutic components of broader treatments that have been previously tested and whose efficacy has been demonstrated separately, such as group psychoeducation (Colom et al., 2003, 2009), family intervention (Reinares et al., 2004, 2008), and functional remediation (Bonnin et al., 2016; Torrent et al., 2013). In addition, emphasis is given on physical health and a module of mindfulness training based on the Mindfulness-Based Cognitive Therapy (Segal, Williams, & Teasdale, 2001) has been incorporated since some evidence suggests the benefits of mindfulness on depressive and anxiety symptoms (Ives-Deliperi, Howells, Stein, Meintjes, & Horn, 2013; Perich, Manicavasagar, Mitchell, & Ball, 2013; Williams et al., 2008) which are common residual symptoms in BD and can negatively affect prognosis. For further details regarding the integrative therapy, see Reinares et al. (2019) and Valls et al. (2020).

Hence, the main aim of the present randomized controlled trial (RCT) was to test the efficacy of this novel integrative approach using psychosocial functioning as the primary outcome variable. Secondary outcomes include reduction in the number of relapses and hospitalizations as well as in depressive, manic, and anxiety symptoms, and an improvement of well-being, quality of life, and cognitive performance. We hypothesized that patients belonging to the integrative approach would have better psychosocial functioning, better well-being, and better quality of life compared to the patients in the control group. They would also show a better course of the disease, in terms of relapses and hospitalizations, a reduction in symptomatology, and better neuropsychological performance compared to the patients included in the control group.

## Methods

### Design

The current RCT examined a very well designed, standardized psychotherapeutic group intervention.

This is a parallel two-armed, single-blind RCT of an integrative approach plus treatment as usual (TAU) *v.* TAU alone. This preliminary study focuses on the pretreatment *v.* post-treatment comparison. The trial protocol contains full details of the study methodology (Valls et al., 2020). The CONSORT guidelines for RCTs (Moher et al., 2010) were followed.

### Participants

The target sample size was established at 132 participants (66 in the intervention group and 66 in the control group). The main variable used to calculate the sample size was the change in psychosocial functioning, which was measured by means of the Functioning Assessment Short Test (FAST). Details are described in Valls et al. (2020). Nevertheless, the trial had to be interrupted indefinitely in February 2020 due to de Covid-19 crisis.

In order to be eligible to be enrolled in the study, participants had to meet the following inclusion criteria: (a) aged between 18 and 60 years old; (b) diagnosis of BD type I or II according to DSM-5 criteria (American Psychiatric Association, APA, 2013); (c) euthymic or with subthreshold symptoms defined as Hamilton Depression Rating Scale (HDRS) <14 (Cordero Villafáfila & Ramos-Brieva, 1986; Hamilton, 1960) and Young Mania Rating Scale (YMRS) <8 (Colom et al., 2002; Young, Biggs, Ziegler, & Meyer, 1978), and (d) the absence of any acute mood episode in the 3 months before the inclusion in the study. Exclusion criteria included: (a) estimated intelligence quotient (IQ) <85; (b) electroconvulsive therapy in the past 6 months or any significant physical or neurologic illness that can affect neuropsychological performance; (c) diagnosis of substance use disorder (SUD) according to DSM-5 (APA, 2013) criteria; (d) inability to understand the purposes of the study, and (e) having had participated in the following psychosocial interventions in the past 2 years, including: psychoeducation, functional remediation, or mindfulness-based interventions.

All eligible participants were recruited between July 2019 and February 2020.

Participant withdrawal was considered when a patient voluntarily discontinued, when they did not attend at least eight intervention sessions (APA, 2013), and/or required psychiatric hospitalization during the group.

The present RCT was conducted in accordance with the ethical principles of the Declaration of Helsinki and Good Clinical Practice. The protocol was approved by the Hospital Clinic Ethics and the Research Board (HCB/2017/0432) and properly registered at clinical trials (www.clinicaltrials.gov; NCT04031560).

### Study procedure

The study was developed at the Barcelona Bipolar and Depressive Disorders Unit at Hospital Clínic, which is part of the Center of Biomedical Research Network for Mental Health (CIBERSAM) (Salagre et al., 2019). Potential participants were informed through the outpatient mental health unit and referrals from mental healthcare professionals. If a participant was interested, he/she was given more detailed information about the study and if he/she finally confirmed willingness and availability, written informed consent was provided. After this, the baseline assessment was performed. Researchers in charge to perform the baseline assessment including the clinical, demographic, functional, and neuropsychological assessment were blind to the treatment allocation. Due to the time required (5 h approximately), in most cases the evaluation was divided into 2 days (2 h 30 min each) with a 10-min break, if required by the participant. All patients underwent the baseline assessment before randomization (details regarding the assessment are described below). Computerized randomization allocated study arms 1:1 with no stratification. MR, EVA and AMA enrolled participants; CMB generated the random allocation sequence, and assigned participants to interventions using Random Allocation Software (version 1.0; Saghaei, 2004) without any restriction (such as blocking or block size). Patients in the active arm underwent the integrative approach as an add-on to pharmacological treatment; while patients in the control group only received TAU, a usual standard pharmacological treatment without additional group sessions. Patients were re-assessed at 3-month follow-up with the same assessment battery used at baseline. The raters (IT, MB, and MPT) who evaluated the outcomes were blind to the assignment to the groups.

### Description of the integrative approach

The adjunctive integrative approach consists of 12 weekly group sessions, 90 min each, implemented in a closed-group setting, over a period of 3 months provided in the outpatient clinic. Four intervention groups were conducted. Each group comprised of between 10 and 14 participants. The integrative approach incorporates different contents including: psychoeducation for patients combined with a session for family members only, and complemented with aspects related to healthy lifestyle, mindfulness training, and strategies for cognitive and functional enhancement. The basis of the approach and the contents of each session have been extensively explained in a published manual (Reinares et al., 2019). The groups were conducted by two psychologists with clinical expertise both in the management of BD and in group interventions (Table 1).

**Table 1.** Session program of the integrative approach

Sessions		Exercises
Session 1	Bipolar disorder: causes and triggers	Drawing a graph with the course of the illness
Session 2	Symptoms of bipolar disorder: early detection of warning signs and early action	Creation of a list of prodromes
Session 3	Treatment of bipolar disorder and therapeutic adherence	
Session 4	Regularity of habits and a healthy lifestyle	Diaphragmatic breathing training Jacobson's progressive muscle relaxation Apps and links to promote monitoring and healthy habits
Session 5	Psychoeducation aimed at family members: family and bipolar disorder	
Session 6	Mindfulness training: automatic pilot *v.* awareness	Raisin exercise (mindful eating) Mindful breathing
Session 7	Mindfulness training: habits of the mind and importance of the body	Three-min breathing space Practice the dynamics of ‘letting go’ Body scan
Session 8	Mindfulness training: thoughts and emotions	Full attention to sounds and thoughts Full attention to emotions
Session 9	Cognitive and functional enhancement: attention and memory	Full attention to the present Story-telling technique Categorization technique
Session 10	Cognitive and functional enhancement: executive functions	Mindful walking Time planning and time management
Session 11	Problem-solving skills training	Problem solving training
Session 12	Assertiveness and communication skills	Role play communication skills

### Description of the TAU group

All the participants in this condition were adult patients attending the outpatient mental health clinic of the Hospital Clinic of Barcelona, diagnosed of BD type I or II according to DSM-5 criteria (American Psychiatric Association, APA, 2013), with euthymic or subthreshold symptoms (HDRS <14, YMRS <8).

They did not receive any additional psychotherapy, just only treated on their regular pharmacological treatment prescription which was based according to the clinical guidelines for the treatment of BD. A semi-structured interview of the Program's protocol based on the Structured Clinical Interview for DSM (APA, 2013) was conducted and complemented with clinical record reviews in order to collect different variables at baseline and after finishing the intervention. Data collection consisted of demographic variables, such as gender, age, years of education, and estimated IQ. Collection of clinical variables, included: chronicity, total number and type of previous episodes, number of hospitalizations, history of psychotic symptoms, bipolar subtype (I or II), family history of affective disorder, number of suicide attempts, and psychiatric medication.

### Outcome measures

#### Primary outcome: psychosocial functioning

It was measured by means of the FAST (Rosa et al., 2007). The FAST is an interviewer-administered instrument developed to assess the main difficulties in daily life of patients with BD. It consists of 24-items and provides a total score and also scores on six specific domains which include: autonomy, occupational functioning, cognitive functioning, financial issues, interpersonal relationships, and leisure time. The overall FAST total score ranges from 0 to 72 and higher scores indicate greater disability.

#### Secondary outcomes

*Clinical variables*: To assess depressive symptoms, the HDRS was administered, and the Hamilton Anxiety Rating Scale (HAM-A) for anxiety symptoms. To assess the severity of manic symptoms the YMRS was used. Additionally, number of hospitalizations and number and type of episodes during the 3-month period of the intervention were also recorded.

*Wellbeing and quality of life*: It was assessed through the Spanish version of the WHO (Five) Well-being Index (WHO-5) (WHO, 2004), which has been validated in Spanish for patients with BD (Bonnín et al., 2017). Quality of life was assessed with the Spanish version of Quality of Life in Bipolar Disorder scale (QoL.BD) (Michalak & Murray, 2010; Morgado, Tapia, Ivanovic-Zuvic, & Antivilo, 2015).

*Subjective cognitive deficits* were assessed through the Cognitive Complaints in Bipolar Disorder Rating Assessment (COBRA) (Rosa et al., 2013). This a self-reported questionnaire consisting of the assessment of subjective cognitive difficulties in daily activities experienced by patients with BD. Higher scores in the total score reflect higher levels of subjective cognitive complaints.

#### Neuropsychological assessment

It was evaluated through a comprehensive neuropsychological battery that lasted approximately 180 min. Different cognitive domains were assessed including: (1) estimated premorbid IQ; (2) processing speed; (3) working memory; (4) executive functions; (5) verbal learning and memory; (6) visual memory; (7) attention; and (8) social cognition. Details regarding the tests included in each of these domains are specified elsewhere (Valls et al., 2020).

### Statistical analysis

For the descriptive analyses, independent sample *t* tests were performed in order to describe the baseline characteristics of both samples; these continuous variables were summarized as means and standard deviations (s.d.). For qualitative variables, chi tests were performed to describe the differences between groups and presented as counts and percentages. To ensure that randomization had worked properly, an additional independent sample *t* test was used to determine whether participants randomized to the integrative approach condition differed significantly from the TAU group participants in their baseline levels of FAST scale, which was the primary outcome. Age and years of education were also included.

To analyze the impact of the intervention on functional outcome in the two groups (integrative approach *v.* TAU) repeated-measures analysis of variance (ANOVA) were conducted using participants' scores on the FAST from baseline to post-treatment, using group allocation as an independent factor. Moreover, six additional repeated measures analyses were performed to analyze each domain of the FAST (autonomy, occupational functioning, cognitive functioning, financial issues, interpersonal relationships, and leisure). Within effect sizes (Cohen's *d*) were also calculated to quantify the effect of the intervention for the global score of the FAST, as well as to evaluate the changes in these six domains.

For the remaining secondary outcomes including HDRS, HAM-D, YMRS, WHO-5, QoL.BD, and COBRA, number of hospitalizations, number and type of relapses and neuropsychological variables, repeated-measures ANOVA were conducted using the same procedure detailed above for the primary outcome. To analyze the neurocognitive outcome, different composite scores for the main cognitive areas were calculated which included the following domains: (1) verbal learning and memory; (2) executive functions; (3) attention, (4) processing speed; (5) working memory; (6) visual memory; and (7) social cognition. The variables included in each composite score correspond with the tests to assess the different cognitive areas that have already been specified in Valls et al. (2020). Given that most of the domains assessed involved different tests, their scores were normalized (converted into *z*-scores) and added-up. Higher scores in each composite score indicate better neuropsychological performance. The variables whose scores indicated worse performance [i.e. Trial Making Test (TMT)-A, TMT-B, Wisconsin Card Sorting Test perseverative errors, and the variables in the Continuous Performance Test (CPT-II) commissions, omissions, reaction time, attentiveness, and beta] were multiplied by (−1) in order to be interpreted and added up in the same direction as the remaining variables.

### Handling of missing data

We restricted the analysis to those participants with no missing data on variables of interest (mainly the FAST scale); this method is also called Available Data Only. It assumes that complete cases are like incomplete cases. It also gives unbiased estimates if the reduced sample resulting from list-wise deletion is a random subsample of the original sample.

Data were analyzed using SPSS, version 18.0 and alpha level was set at *p* < 0.05 (two-tailed).

## Results

### Sample description

**Fig. 1.**
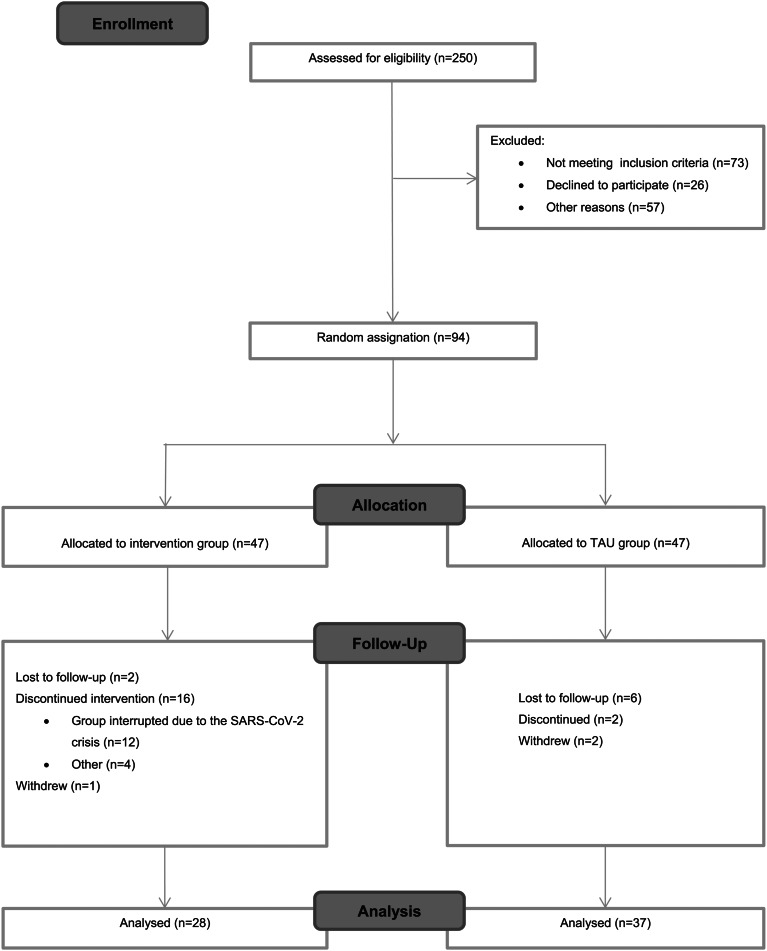
CONSORT flowchart.

Figure 1 shows recruitment and retention from baseline to post-treatment. In total, 250 patients were screened for this study; however, for different reasons only 94 were randomized at baseline. The primary reason for no eligibility was not meeting inclusion criteria (*n* = 73; 29.2%), followed by not being able to participate for incompatibility work times (*n* = 57; 22.8%); and finally, not interested to participate in the study (*n* = 26; 10.4%).

Regarding the attrition rates, of the 47 participants enrolled in the intervention group at baseline, 28 completed the intervention yielding a 59.6% completion rate. The high attrition rate in this group might be explained because the last group (*n* = 12; 25.5%) which started in February 2020, had to be interrupted due to the SARS-CoV-2 crisis, forcing us to stop both the group and the trial indefinitely. The completion rate without including the last group would have yielded up to 80% (*v*. 59.6%). Besides this exceptional situation, a total of *n* = 4 (8.5%) discontinued for other reasons including disease relapse or not attending enough sessions.

The participants' clinical and demographic characteristics at baseline are summarized in Table 2. There were no significant differences between two groups regarding age (*t* = 0.27; *p* = 0.78), gender (chi = 2.15; *p* = 0.14) or estimated premorbid IQ (*t* = 0.14; *p* = 0.89). Furthermore, no significant differences were found in the primary outcome at baseline measured with the FAST (*t* = −0.72; *p* = 0.47). Significant differences between both groups were found in the HDRS total score (*t* = −3.5; *p* < 0.01), YMRS total score (*t* = −2.79; *p* < 0.01), and chronicity (years of illness) (*t* = 0.67; *p* = 0.01), specifically, patients in the TAU group scored higher.

**Table 2.** Participants' clinical and demographic characteristics at baseline

	Integrative approach (*n* = 28) Mean (s.d.)	TAU group (*n* = 37) mean (s.d.)	*t* (*p*)
Age	47.61 (7.34)	45.81 (9.92)	−0.81 (*p* = 0.42)
Years of education	16.14 (4.18)	15.46 (3.28)	−0.74 (*p* = 0.46)
Estimated IQ	112.19 (10.14)	113.11 (9.38)	0.38 (*p* = 0.71)
FAST total score	23.18 (10.76)	22.95 (14.03)	−0.073 (*p* = 0.94)
HDRS total score	6.70 (4.12)	3.81 (3.22)	−3.15 (*p* < 0.01)
YMRS total score	2.32 (2)	1.05 (1.67)	−2.79 (*p* < 0.01)
HAM-D total score	9.64 (7.71)	6.76 (7.65)	−1.5 (*p* = 0.14)
Chronicity (years of illness)	21.61 (8.18)	16.03 (8.42)	−0.67 (*p* = 0.01)
Total number previous episodes	8 (5.65)	9.29 (8.09)	0.72 (*p* = 0.48)
Number of previous manic episodes	1.14 (1.46)	2.15 (2.65)	1.89 (*p* = 0.07)
Number of previous depressive episodes	4.64 (3.62)	5.62 (7.88)	0.64 (*p* = 0.52)
Number of previous hypomanic episodes	2.25 (2.73)	1.41 (1.99)	−1.39 (*p* = 0.17)
Number of previous hospitalizations	1.29 (1.46)	1.97 (2.04)	1.51 (*p* = 0.14)
	*n* (%)	*n* (%)	Chi (*p*)
Gender (women)	12 (42.9)	22 (59.5)	1.76 (*p* = 0.18)
Diagnosis (type I)	15 (53.6)	26 (70.3)	1.9 (*p* = 0.17)
Lifetime psychotic symptoms (yes)	17 (60.7)	20 (54.1)	0.29 (*p* = 0.59)
Suicide attempts (yes)	9 (32.1)	8 (21.6)	0.91 (*p* = 0.34)
Family history of affective disorder (yes)	20 (76.9)	17 (45.9)	6.05 (*p* = 0.14)
Antipsychotic (yes)	20 (71.4)	24 (64.9)	0.31 (*p* = 0.58)
Antidepressants (yes)	18 (64.3)	17 (45.9)	2.16 (*p* = 0.14)
Lithium (yes)	14 (50)	20 (54.1)	0.12 (*p* = 0.75)
Anticonvulsants (yes)	16 (57.1)	20 (54.1)	0.06 (*p* = 0.8)
Benzodiazepine (yes)	9 (32.1)	16 (43.2)	0.83 (*p* = 0.36)

### Primary outcome: changes of the FAST: from baseline to post-treatment

The repeated-measures analyses comparing baseline and post-treatment revealed a significant group × time interaction in favor of the patients who received the integrative approach compared to the TAU group (Pillai's trace = 0.10; *F*_(1,63)_ = 7.1; *p* = 0.01) indicating that patients who received the active intervention improved their psychosocial functioning from 23.2 (10.7) at baseline, to 17.1 (9.3) after finishing the intervention post-treatment. Patients in the TAU group remained practically the same: 23.2 (14) at baseline, and 23.4 (14.7) at the end of the intervention.

In accordance with the CONSORT 2010 guidelines on baseline data, analyses adjusting for any baseline differences in demographic or clinical characteristics between the groups would be performed in case of imbalances between the groups for variables with effects on the primary outcome (Moher et al., 2010). Given that patients in both groups differed in terms of the HDRS and YMRS total scores at baseline and for chronicity (years of illness) (see Table 2), we performed an additional repeated measures analysis controlling for these confounding variables. After this, the group × time interaction in favor of patients who received integrative approach was still significant (Pillai's trace = 0.11; *F*_(1,57)_ = 6.9; *p* = 0.011).

Regarding the domains of the FAST, two out of the six domains reached statistical significance, which were: the cognitive functioning domain (Pillai's trace = 0.11; *F*_(1,63)_ = 8.4; *p* = 0.005) and leisure time (Pillai's trace = 0.09; *F*_(1,63)_ = 6.7; *p* = 0.012). Both domains remained significant after correcting for the above-mentioned confounding variables. Particularly, patients in the active arm reduced difficulties in the cognitive domain, on average, almost two points: from 4.3 (3.3) at baseline, to 2.5 (2.3) post-treatment (Pillai's trace = 0.25; *F*_(1,57)_ = 19.1; *p* < 0.0015). Additionally, patients in the integrative group improved their functioning in the leisure time domain from 2.4 (1.4) to 1.7 (1.28) (Pillai's trace = 0.11; *F*_(1,57)_ = 7.15; *p* = 0.01).

**Fig. 2.**
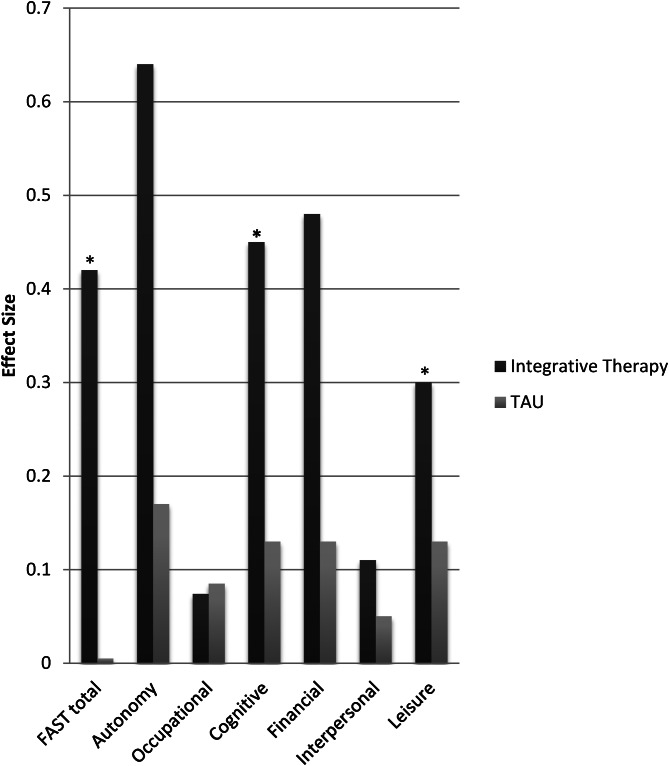
Within-groups Cohen's *d* effect size in the FAST total score and the specific domains.

Significant Cohen's *d* effect sizes for the total score of the FAST and for the different domains are displayed in Fig. 2.

### Secondary outcomes

#### Symptoms and subjective measures

No significant differences were found in any of the secondary outcomes except for depressive symptoms (HDRS): a significant group × time interaction indicated that patients who received the integrative approach improved significantly in depressive symptoms (Pillai's trace = 0.08; *F*_(1,62)_ = 5.6; *p* = 0.02) from 6.7 (4.21) at baseline to 5.04 (3.3) to post-treatment. No significant differences in group × time interaction were found in anxiety symptoms (HAM-A), manic symptoms (YMRS), subjective cognitive deficits (COBRA), wellbeing, and quality of life (WHO-5, QoL.BD).

#### Relapses and hospitalizations

As for the number of relapses and hospitalizations, no patients were hospitalized or presented manic episodes during the 3-month period that lasted the present study. As regards the number of episodes of depression, patients who received the intervention did not suffer any depressive episode, while three patients in the TAU group suffered a depressive episode, increasing the group mean from 5.6 (7.9) at baseline to 5.7 (8.04) at post-treatment; nevertheless, this difference was not statistically significant (Pillai's trace = 0.04; *F*_(1,60)_ = 2.6; *p* = 0.11). Regarding hypomanic episodes, patients in the TAU group did not suffer any relapse, while one patient in the first intervention group of the integrative approach suffered one relapse, increasing the group mean from 2.25 (2.7) at baseline to 2.29 (2.8) after the treatment period, but the difference between groups was not statistically significant (Pillai's trace = 0.02; *F*_(1,60)_ = 1.22; *p* = 0.27).

#### Neuropsychological outcomes

After grouping the variables into six different composite scores (attention, executive functions, verbal learning and memory, processing speed, visual memory, working memory, and social cognition), no significant differences in group × time interaction were found in any of these domains.

Changes regarding these above-mentioned secondary outcomes are displayed in Table 3.

**Table 3.** Between-group differences in the secondary outcomes at baseline and after treatment

	Integrative therapy		TAU			
	Baseline mean (s.d.)	Follow-up mean (s.d.)	Baseline mean (s.d.)	Follow-up mean (s.d.)	*F*_(df)_	*p*
Clinical outcomes						
Depressive symptoms (HDRS)	6.7 (4.1)	5.0 (3.3)	3.8 (3.3)	4.5 (4.3)	*F*(1,62) = 5.6	0.02
Manic symptoms (YMRS)	2.3 (2.0)	2.0 (2.3)	1.05 (1.6)	1.22 (2.0)	*F*(1,63) = 0.7	0.40
Anxiety symptoms (HAM-A)	9.64 (7.7)	6.18 (6)	6.76 (7.7)	5.70 (5.3)	*F*(1,63) = 1.9	0.17
HAM-A psychic	5.50 (3.74)	3.82 (3.14)	4.41 (4.37)	3.76 (3.56)	*F*(1,63) = 1.04	0.31
HAM-A somatic	4.14 (4.59)	2.36 (3.13)	2.35 (3.59)	1.95 (2.4)	*F*(1,63) = 2.6	0.11
Well-being (WHO-5)	11.74 (6)	12.74 (5.9)	13.81 (5.4)	13.97 (5.7)	*F*(1,57) = 0.5	0.47
Quality of life (QoL.BD)	15 836 (29.5)	154.2 (27.9)	160.7 (38.1)	162.6 (31.59)	*F*(1,53) = 0.8	0.37
Subjective cognitive complaints (COBRA)	22 (7.6)	21 (8.5)	21.78 (10.6)	19.75 (9.29)	*F*(1,56) = 0.6	0.45
Neuropsychological outcomes (composite scores)						
Attention	1.46 (3.23)	0.35 (3.95)	−0.15 (4.66)	−0.27 (5.46)	*F*(1,59) = 1.12	0.29
Executive functions	0.13 (3.03)	0.13 (3.49)	0.2 (4.75)	−0.19 (4.5)	*F*(1,58) = 0.24	0.63
Working memory	0.29 (1.08)	0.09 (1.11)	0.06 (1.08)	−0.07 (0.93)	*F*(1,59) = 0.1	0.75
Processing speed	0.14 (0.8)	0.19 (0.57)	0.16 (1.08)	−0.14 (1.23)	*F*(1,59) = 1.81	0.18
Verbal learning and memory	0.67 (4.89)	0.45 (4.76)	0.53 (3.87)	−0.29 (4.79)	*F*(1,59) = 0.79	0.38
Visual memory	0.18 (0.88)	0.16 (1.1)	−0.07 (0.99)	−0.04 (0.88)	*F*(1,58) = 0.04	0.83
Social cognition	−0.03 (2.85)	0.07 (2.17)	−0.96 (2.67)	0.11 (2.15)	*F*(1,31) = 1.72	0.2
Relapses and hospitalizations outcomes						
Depressive episodes	4.64 (3.6)	4.64 (3.6)	5.6 (7.9)	5.7 (8.05)	*F*(1,60) = 2.62	0.11
Hypomanic episodes	2.25 (2.7)	2.28 (2.8)	1.41 (1.99)	1.41 (1.99)	*F*(1,60) = 1.22	0.27

## Discussion

The present RCT is a well-designed, standardized psychotherapeutic group intervention and the results suggest that the participation in an adjunctive, comprehensive psychotherapeutic program, combining the main components of different approaches (including psychoeducation, mindfulness, and functional remediation), was effective at improving the global psychosocial functioning, and also at improving two particular areas of functioning, including the cognitive and leisure domains of the FAST scale with moderate effect sizes. These results remained significant even after controlling for confounding variables. Regarding the secondary outcomes, a significant reduction in the levels of subsyndromal depressive symptoms was found. Hence, the present results suggest that the integrative approach seems to improve general psychosocial functioning and residual depressive symptoms in patients suffering from BD.

To the best of our knowledge, this is one of the few studies testing the efficacy of a brief multicomponent psychological intervention using psychosocial functioning as the main outcome variable. Even though over the past decade there has been a growing interest in studying psychosocial functioning, there is still a lack of studies testing this particular area (Solé & Vieta, 2020). Some of the first RCT that used psychosocial functioning as the main outcome variable include studies from our group. Specifically, Torrent et al. (2013) and Bonnin et al. (2016) tested the efficacy of functional remediation, a 21-session rehabilitation program, addressed to improve cognition and psychosocial functioning in euthymic patients with BD. The program demonstrated its efficacy at enhancing functioning both at immediately after intervention (Torrent et al., 2013) and at 12-month follow-up (Bonnin et al., 2016). Nevertheless, it is worth mentioning that this intervention was designed for chronic patients (patients in the late stages of the illness), with moderate to severe functional impairment and/or with cognitive problems (Bonnin et al., 2014; Martínez-Arán, 2011). Moreover, it lasts up to 6 months, in contrast with the integrative approach which lasts only 3 months and this latter one can be implemented in earlier stages of the illness. Another study (Fiorillo et al., 2015) tested the efficacy of a psychoeducational family intervention with the objective to improve functioning in patients with BD type I, finding a positive effect of the intervention on patients' disability and on family burden. Haffner et al. (2017) also described that the metacognitive training, a multimodal intervention with a total of eight sessions, could enhance psychosocial functioning in euthymic patients, with significant improvement of several areas of the FAST scale including: autonomy, occupational, cognitive, and interpersonal domains; however, this latter report was a pilot study without randomization. This growing interest in designing interventions to enhance functioning is not surprising, since BD is considered among the most burdensome mental disorders (Whiteford et al., 2013). Pharmacological therapy alone might not be enough to tackle these functionality problems and, in fact, it has been suggested that prior disability status could be associated with worse pharmacological response (Deckersbach et al., 2016), highlighting the key role that psychosocial functioning may exert over the course of BD and on the efficacy of the prescribed drugs. Hence, there is a need to continue developing and testing new psychological interventions to optimize the treatment and course of these patients (Deckersbach et al., 2016). In this regard, the present intervention, the integrative approach, has shown to improve psychosocial functioning in only 3 months, including the global functioning and two specific domains: cognitive functioning and leisure.

The changes found in the cognitive domain of the FAST deserve further explanation. This area is supposed to measure the cognitive difficulties that some patients present; its scoring is not only based on patients' report, but also on clinical judgment. Thus, is not purely subjective (what patients say) but it is neither as objective as the neuropsychological tests can be, since clinicians are not foolproof. Together with this result, it is also worth mentioning that no significant changes were found in the COBRA, which is a scale that measures the subjective cognitive complaints, and no significant changes were found in any of the cognitive domains assessed with objective measures (neuropsychological battery) either. There might be different explanations for these above-mentioned results: one could be that the changes in the cognitive domain of the FAST reflect the difficulty that the clinicians have distinguishing some signs and symptoms of the illness such as cognitive impairment and depressive symptoms; for instance, the clinician can interpret that having difficulties to keep concentration either as a depressive symptom or as cognitive impairment. Another possible explanation is that the study was not powered enough to detect the subtle changes that may have occurred in neurocognition or that the time of the treatment (only 3 months long) is still too short to detect significant differences. In this regard, one study (Bonnin et al., 2016) suggested that the cognitive changes might take some more time to consolidate (up to 6 months from baseline) after an intervention. It is also worth mentioning that the module of the cognitive/functional enhancement might be too short to produce significant cognitive changes, since it includes only four sessions; it is possible that a more intensive practice is needed to produce significant and long-lasting changes in this area. Finally, the fact that the subjective complaints, measured by the means of the COBRA, did not change might not be surprising, since it is well established the discrepancy existing between objective and subjective complaints in both BD (Martínez-Arán et al., 2005; Miskowiak et al., 2016) and unipolar depression (Petersen, Porter, & Miskowiak, 2018).

Concerning the improvement detected in the leisure domain of functioning, it could be explained in part by the mindfulness exercises, given that such training contributes to decreasing rumination and promotes exposure to experiences by inhibiting avoidance behaviors (Parsons, Dreyer-Oren, Magee, & Clerkin, 2019). That is, changing the way the activities are faced by paying attention to the experiences on purpose, focusing on the present and non-judgmentally, increasing awareness of what is happening moment by moment. In this line, previous studies have found an association between Mindfulness-based Cognitive Therapy (MBCT) and experiencing momentary positive emotions and greater appreciation and response to pleasant activities of daily life (Geschwind, Peeters, Drukker, van Os, & Wichers, 2011). The authors of the reported article consider that these changes are unlikely to be due solely to a decrease in depressive symptoms, given the role of positive emotions in resilience against depression, and may contribute as protective effects against depressive relapse (Geschwind et al., 2010, 2011). It might be possible that a similar process occurred in the patients of this sample, since the improvement in leisure remained even after controlling for depressive symptoms. Another possible explanation includes the fact that the implementation of the therapy was in a closed-group format, and this could have favored interpersonal interactions by increasing contact with others and motivation to carry out social activities. There is also a specific session promoting healthy life styles and healthy habits, which is implemented at the very beginning of the intervention. If most of the patients engaged the recommendations given in that session and emphasized during the group meetings, it could also explain the positive changes observed in this domain favoring the engagement in certain daily self-care and pleasant activities such as those related to physical exercise, social life, and hobbies. Despite these results, it is necessary that future studies confirm these outcomes with larger samples and in the long term with follow-up assessments in order to be able to verify if the results are maintained over time.

As regards the reduction in the HDRS scores could be explained as a result of the synergy between the different components of the program. Indeed, mindfulness-based cognitive therapy has been associated with a reduction of depressive symptoms in a recent review (Xuan et al., 2020). Skills such as emotional regulation, together with other sessions related to psychoeducation, which also emphasize coping skills to deal with symptoms by correcting false beliefs and, in case of depressive signs, promoting behavioral activation (increasing daily pleasant activities and social relationships through improving social skills), self-care (healthy habits, physical exercise, and regularity in sleep patterns), and the fact of attending weekly to a group intervention, could have increased the exposure to response-contingent positive reinforcement, and as a consequence the reduction of these residual depressive symptoms. It is possible that all the components tackle, indirectly, the engagement and commitment in meaningful behaviors that maximize exposure to natural reinforcements, and as a consequence, a reduction in depressive residual symptoms (Lejuez, Hopko, Acierno, Daughters, & Pagoto, 2011). The knowledge and acceptance of BD, as well as the optimization of strategies to better manage the illness and stressful situations may play a crucial role.

Finally, no significant differences were found in the remaining secondary variables, including number and type of relapses, changes in quality of life and in well-being. These negative results might be explained, partially, due to the brief intervention (only a 3-month intervention) and a longer follow-up period might be needed to detect the efficacy of the integrative approach at preventing relapses, as well as to detect significant changes in subjective measures related to patients' wellbeing.

Nevertheless, the present results point out the effectiveness of the program at improving psychosocial functioning in the post-treatment assessment. Future studies are required to confirm these current results considering the long-term effects with follow-up both at 6 and at 12 months.

Given these preliminary positive results, future studies could consider to broaden the inclusion criteria so that more severe patients could benefit from this comprehensive approach, it might be also considered to complement the integrative approach with additional psychotherapy individual treatment, at least in the most severe patients.

### Limitations

The current preliminary results of the RCT present some limitations that deserve to be mentioned. First, the lack of a third condition arm as an active control treatment does not allow us to control for placebo-like effects, limiting the clinical interpretation of the study. We faced difficulties in patient recruitment, as many of them were excluded from the study as they did not meet the inclusion/exclusion criteria stated in the protocol study. Hence, it is necessary that future studies include an active control group for comparison in order to verify the present results. Second, since the integrative approach is a multi-component program, it is difficult to know which components are effective and responsible of the improvements detected; on the contrary, this type of program might be more attractive for patients and may increase adherence since they include a variety of modules and tackle different areas that promote self-care and well-being. Third, there has been more dropouts than expected, mainly due to the current Covid-19 pandemic crisis (SARS-CoV-2) that forced us, as many other research groups worldwide (Stefana et al., 2020; Vieta, Pérez, & Arango, 2020), to stop both the whole trial and the last intervention group in March 2020; as a consequence, the sample size was reduced drastically, limiting the power to detect significant changes in the outcome variables; despite this, statistically and clinically significant changes have been detected in the main outcome variable. The impact of the Covid-19 pandemic on patients with BD is disrupting both public and private mental health services, and most patients are unable to access outpatient care. Thus, the pandemic forces a rethinking of how best to improve access to and implementation of BD-specific psychological and psychiatric intervention services (Stefana et al., 2020). If face-to-face groups are not possible, mixed therapies that combine face-to-face and online, or online alternatives should be taken into consideration. Fourth, additional analyses to control for confounding variables were performed for the primary outcomes but not for secondary outcomes such as depressive symptoms or relapses. Fifth, another limitation was the difficulty in monitoring the pharmacological changes during the intervention, although at the beginning of the study the two groups were comparable in the prescribed pharmacological treatment. Sixth, we have only measured depressive symptoms at baseline and once at follow-up. Finally, the sample has been recruited at a mental health center specialized in BD, thus it is unknown if the present results are applicable to patients from other centers who present less severe courses of the illness.

## Conclusions

The results of this RCT show the efficacy of the integrative approach at improving the general psychosocial functioning and two specific domains (cognitive functioning and leisure time), which were maintained even after controlling for confounding variables. The moderate effect sizes, although preliminary due to the small sample size, are also promising. An improvement in depressive residual symptoms was also found in favor of the patients who received the integrative approach. Hence, the integrative approach represents a cost-effective psychological intervention, short and feasible, that could be implemented in centers with few resources. Psychological treatments for BD have evolved beyond early versions, whose main objectives were focused on increasing pharmacological adherence and relapse prevention. In this regard, the integrative approach represents an effort to tackle different areas in which patients also present urgent needs but without foregoing the patient's experience and preferences in order to increase patient satisfaction and adherence to the treatment. Furthermore, the inclusion criteria of the patients to whom the intervention was offered were less restrictive (i.e. residual symptoms and comorbidities except for SUD) than those of previous clinical trials in order to provide it to a larger number of subjects which would be more representative of the population with BD.

Future studies should confirm the present results with larger samples, involving not only highly specialized centers and also considering the effects found in the long term, with follow-ups at 6 and 12-months. Along with this, the identification of predictors of response to treatment (Reinares et al., 2020) could also contribute to personalize the treatment of patients with BD.

## Data Availability

The data sets used and/or analyzed accompanying this document are made available from the corresponding author upon a reasonable request.

## Supplementary material

For supplementary material accompanying this paper visit https://doi.org/10.1017/S0033291721001057. click here to view supplementary material
